# Heterogeneity of *Borrelia burgdorferi* Sensu Stricto Population and Its Involvement in *Borrelia* Pathogenicity: Study on Murine Model with Specific Emphasis on the Skin Interface

**DOI:** 10.1371/journal.pone.0133195

**Published:** 2015-07-21

**Authors:** Aurélie Kern, Gilles Schnell, Quentin Bernard, Amandine Bœuf, Benoît Jaulhac, Elody Collin, Cathy Barthel, Laurence Ehret-Sabatier, Nathalie Boulanger

**Affiliations:** 1 EA7290: Virulence bactérienne précoce: groupe Borréliose de Lyme, Facultés de médecine et de pharmacie, Université de Strasbourg, Strasbourg, France; 2 Laboratoire de Spectrométrie de Masse BioOrganique, Institut Pluridisciplinaire Hubert Curien, Université de Strasbourg, CNRS, UMR7178, Strasbourg, France; 3 Division of Geographic Medicine and Infectious Diseases, Tufts Medical Center, Boston, Massachusetts, United States of America; University of Kentucky College of Medicine, UNITED STATES

## Abstract

Lyme disease is a multisystemic disorder caused by *B*. *burgdorferi* sl. The molecular basis for specific organ involvement is poorly understood. The skin plays a central role in the development of Lyme disease as the entry site of *B*. *burgdorferi* in which specific clones are selected before dissemination. We compared the skin inflammatory response (antimicrobial peptides, cytokines and chemokines) elicited by spirochete populations recovered from patients presenting different clinical manifestations. Remarkably, these spirochete populations induced different inflammatory profiles in the skin of C3H/HeN mice. As spirochete population transmitted into the host skin is heterogeneous, we isolated one bacterial clone from a population recovered from a patient with neuroborreliosis and compared its virulence to the parental population. This clone elicited a strong cutaneous inflammatory response characterized by MCP-1, IL-6 and antimicrobial peptides induction. Mass spectrometry of this clone revealed 110 overexpressed proteins when compared with the parental population. We further focused on the expression of nine bacterial surface proteins. *bb0347* coding for a protein that interacts with host fibronectin, allowing bacterial adhesion to vascular endothelium and extracellular matrix, was found to be induced in host skin with another gene *bb0213* coding for a hypothetical protein. These findings demonstrate the heterogeneity of the *B*. *burgdorferi* ss population and the complexity of the interaction involved early in the skin.

## Introduction

Lyme disease, a zoonosis caused by the *Borrelia burgdorferi* sensu lato (sl) group is the most common arthropod-borne disease in the Northern Hemisphere. Among the different *B*. *burgdorferi* sl species, *B*. *burgdorferi* ss sensu stricto (ss) is a pathogenic species present in both the United States and Europe. Lyme disease induces a multisystemic disorder typically characterized first by a skin inflammation, the erythema migrans at the site of *Borrelia* inoculation in human. After hematogenous dissemination systemic manifestations (eg, cardiac, articular, neurological and cutaneous) are observed [1].

The factors responsible for *Borrelia* organotropism are not known. In a mouse model, the skin has been shown to constitute a key interface where the spirochetes multiply before disseminating to target organs [2]. To better evaluate the role of the vertebrate host skin in the transmission, we studied different *B*. *burgdorferi* isolates recovered from patients with distinct clinical manifestations. The skin protects itself against infections by innate immunity and is important to maintain homeostasis [3]. Recently the role of the skin has been re-evaluated in Lyme borreliosis with specific emphasis on the role of OspC in the transmission [4–6] and as a potential filter in *B*. *burgdorferi* populations [7]. We selected genes of innate immunity (antimicrobial peptides, chemokines and cytokines) and compared the skin inflammatory response elicited by human isolates of *B*. *burgdorferi* in a mouse model, as the immune pressure in the skin might drive certain *Borrelia* populations towards specific organs.

Spirochetes refer to distinct species and genomic groups and differ in their pathogenicity [8]. Two main genetic markers, the outer-surface protein C (*ospC*) type and the 16S-23S rRNA intergenic spacer type (RST) can be used to distinguish strains of *B*. *burgdorferi* ss allowing the strains to correlate the severity of their clinical manifestations [9]. Three RSTs have been defined: RST1 genotype is correlated with hematogenous dissemination and severe clinical signs, RST3 isolates are considered as non-disseminating strains, and RST2 isolates as intermediate. Within the former marker, 21 OspC groups have been initially defined [10]. In patients, hematogenous dissemination is mainly associated with types A, B, I and H among the 16 actual OspC genotypes [11]. These genetic markers are currently the best to define specific clinical isolates of *B*. *burgdorferi* [12]. However, they are not sufficient to determine the outcome of an infection initiated by a specific *Borrelia* strain and species. The situation is complicated because of the biodiversity of *B*. *burgdorferi* strains in ticks and tissues [7] and the differing disseminating profiles in clones isolated from one specific *Borrelia* population [13]. Clearly, a selection occurs but the factors that govern it remain to be identified.

In this study we compared the tissue distribution and the inflammatory response of different clinical isolates (also referred to as pathotypes) belonging to different RST groups. Considering that the skin could act as a filter to select specific clones, we selected a specific clone of the 297 strain because of its cerebral tropism. We compared its dissemination and inflammatory pattern to its parental strain. Although these two strains were RST2, they exhibited different inflammatory profiles in the skin. Proteomic analyses on the clone showed that 110 proteins were overexpressed compared to the parental population *in vitro*. Among these, we selected 9 cell envelope proteins and measured their gene expression in mouse skin during the early transmission of the parental 297 strain or in the selected clone 297/4. We propose that the skin, by virtue of its immunity, constitutes an essential organ that selects *B*. *burgdorferi* clones to persist locally or to disseminate. The different factors contributing to this phenomenon remain to be precisely identified. However, we found that the host factors, defensin mBD14 and chemokine MCP-1, are both involved in vascular permeability and angiogenesis, as potential factors participating in this mechanism. Bacterial proteins interacting with host extracellular matrix are also possible candidates as factors facilitating tissue persistence or dissemination of *B*. *burgdorferi*.

## Results

### Transmission and tissue distribution of different pathotypes of *B*. *burgdorferi ss* in mice

All strains studied exhibited a similar pattern of dissemination. *B*. *burgdorferi* was detected at day 3 by PCR or by skin culture at the site of inoculation for all strains. They disseminated rapidly to the ankle, the heart and the bladder where they were first detected at day 5 or 7. The ear, which corresponds to the skin at distance from the inoculation site was the last one to be colonized (Tables 1 and 2). We observed for all strains tested a lack of detection of the bacteria within the skin 24 hours after inoculation. We attribute this result to the PCR or culture detection limit. All mice seroconverted to *B*. *burgdorferi* antigens 15 days after bacterial inoculation (data not shown). Weekly assessments of arthritis were performed by measuring the thickness of both ankles with a metric caliper. Ankle swelling provided an indication of arthritis severity, but no significant difference was observed among the strains tested (data not shown). The blood culture was also tested and was positive only transiently at around day 5 or 7, with no significant difference between the strains (data not shown).

**Table 1:** Kinetic of tissue distribution in mouse of *Borrelia burgdorferi* ss strains isolated from skin manifestations. Tissue distribution of different strains of *Borrelia burgdorferi* ss. Bacterial distribution was measured by culture (heart, skin at inoculation site and ear) or by PCR (bladder, ear and skin at the inoculation site) and by both methods for the ankle at different time points after intradermal injection of 10^3^ *B*. *burgdorferi*. Five mice were analyzed for each time point. RST: rRNA intergenic spacer type; h: hours and d: days. EM: Erythema migrans; MEM: Multiple Erythema Migrans.

	Tissue distribution of strains isolated from skin manifestations									
	Pbre (EM)—RST1					MR726 (MEM)–RST3				
	skin at inoculation site	heart	bladder	ankle	ear	skin at inoculation site	heart	bladder	ankle	ear
24h	0/5	0/5	0/5	0/5	0/5	0/5	0/5	0/5	0/5	0/5
3d	3/5	0/5	0/5	0/5	0/5	5/5	0/5	0/5	0/5	0/5
5d	5/5	1/5	0/5	1/5	0/5	5/5	2/5	1/5	2/5	0/5
7d	5/5	1/5	4/5	4/5	0/5	5/5	5/5	5/5	5/5	0/5
15d	5/5	5/5	5/5	5/5	5/5	5/5	5/5	5/5	5/5	5/5
30d	5/5	5/5	5/5	5/5	5/5	5/5	5/5	5/5	5/5	5/5

**Table 2:** Kinetic of tissue distribution in mouse of *Borrelia burgdorferi* ss strains isolated from cerebrospinal fluid. Tissue distribution of different strains of *Borrelia burgdorferi* ss. Bacterial distribution was measured by culture (heart, skin at inoculation site and ear) or by PCR (bladder, ear and skin at the inoculation site) and by both methods for the ankle at different time points after intradermal injection of 10^3^ *B*. *burgdorferi*. Five mice were analyzed for each time point. (CSF): Cerebro-Spinal Fluid. RST: rRNA intergenic spacer type; h: hours and d: days.

	Tissue distribution of strains isolated from cerebrospinal fluid									
	1808/03 – RST1					297 – RST2				
	skin at inoculation site	heart	bladder	ankle	ear	skin at inoculation site	heart	bladder	ankle	ear
24h	0/5	0/5	0/5	0/5	0/5	0/5	0/5	0/5	0/5	0/5
3d	5/5	0/5	0/5	0/5	0/5	5/5	1/5	0/5	0/5	0/5
5d	5/5	0/5	1/5	0/5	0/5	5/5	1/5	0/5	2/5	0/5
7d	5/5	2/5	5/5	5/5	1/5	5/5	4/5	5/5	5/5	2/5
15d	5/5	5/5	5/5	5/5	5/5	5/5	5/5	5/5	5/5	5/5
30d	5/5	5/5	5/5	5/5	5/5	5/5	5/5	5/5	5/5	5/5

### *B. burgdorferi* pathotype quantification and measure of the inflammation in the skin at the inoculation site

**Fig 1:**
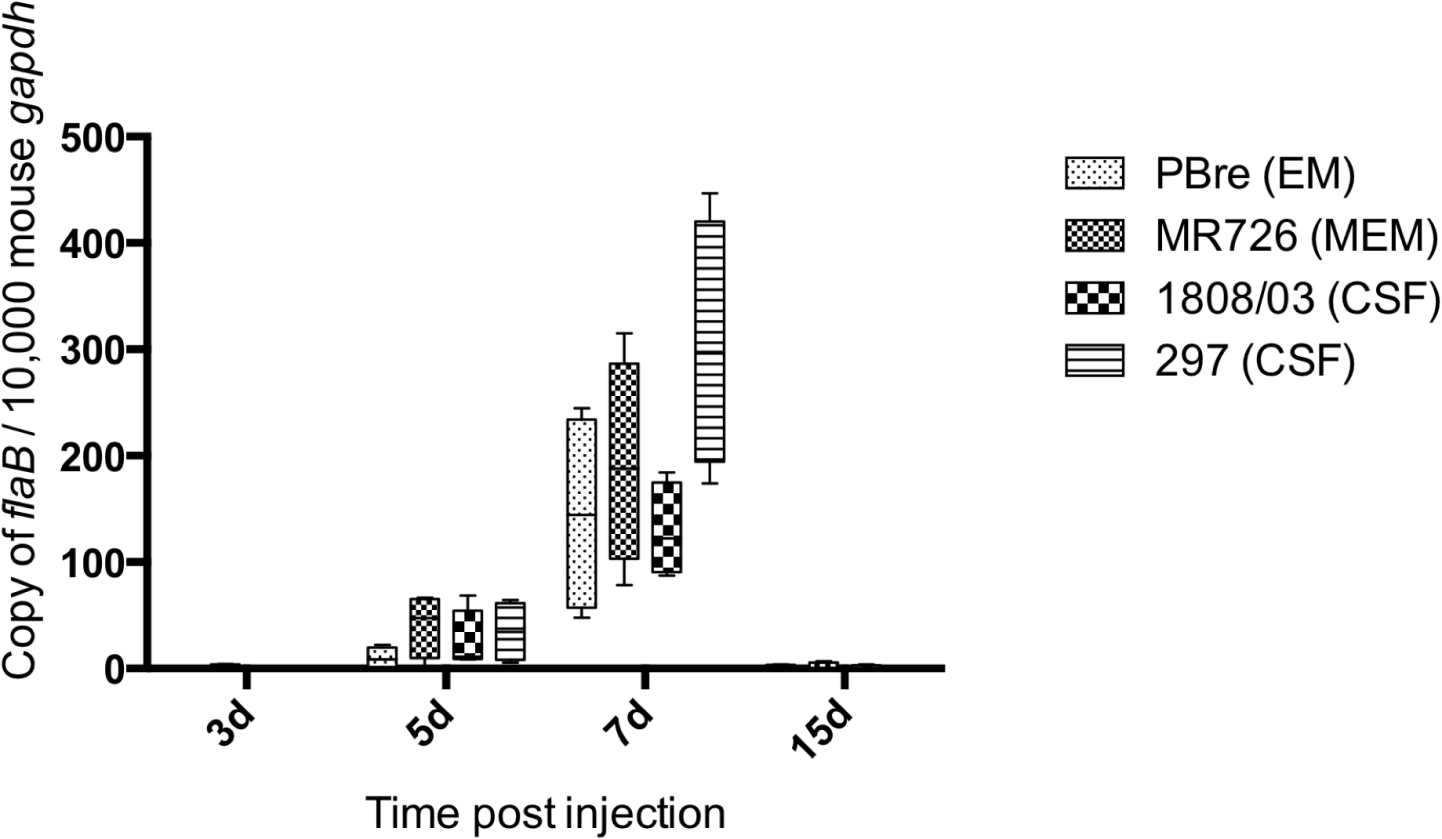
Bacterial loads in the skin. The spirochetal burden in the skin at the inoculation site was measured by quantitative PCR for the *B*. *burgdorferi flaB* gene and normalized to copies of mouse *gapdh*. Values represent relative expression + SD of three independent experiments. Values between strains were not statistically different (two-way ANOVA test using the Sidak-Bonferroni method). (d: days).

We measured the bacterial load in the skin since it was shown previously that the skin is a site of *B*. *burgdorferi* multiplication [14,15]. The bacteria all multiplied intensively at day 7 post-infection, with no significant difference in the dissemination kinetics observed among the strains tested (Fig 1).

**Fig 2:**
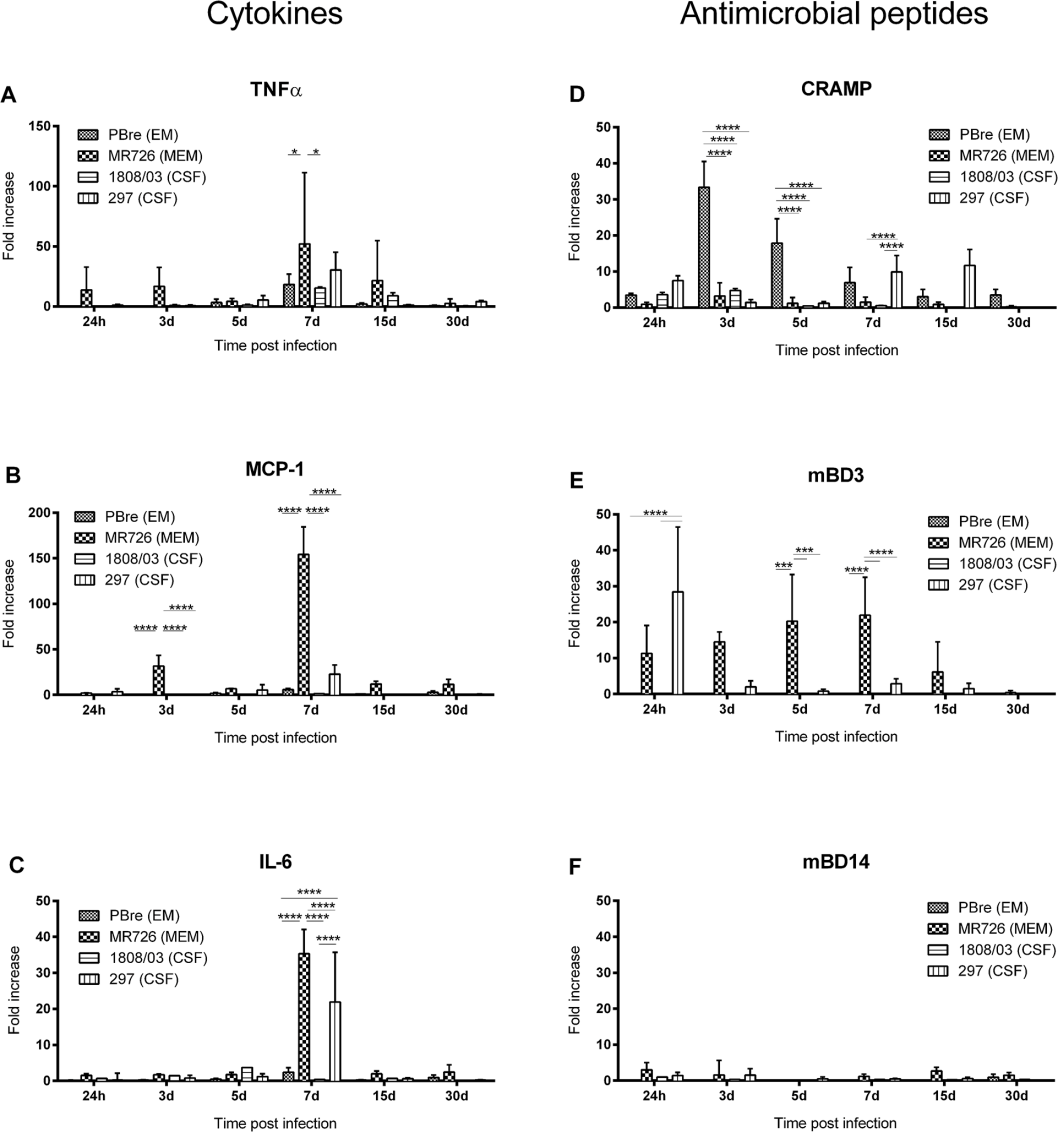
Inflammatory profiles of the different strains of *B*. *burgdorferi* ss. Levels of transcripts were measured by RT-qPCR from the skin at the inoculation site. The values were calculated using the 2-delta delta Ct method after normalization with *gapdh*. (h: hours, d: days). Two-way ANOVA was used to analyze the data. At least three mice were analyzed for each time point.

We showed previously that *B*. *burgdorferi* ss N40 elicited different inflammatory genes of innate immunity in the skin. We tested whether the different pathotypes induced similar genes in the skin. We observed the induction of pro-inflammatory molecules (Fig 2): TNF-α, IL-6 and the chemokine MCP-1. For all of them, a peak induction of TNF-α and/or MCP-1 was observed at day 7 post-infection. Interestingly, MR726 strain isolated from a human MEM lesion induced the strongest inflammatory profile in mouse skin with a peak of MCP-1 (150 fold) at day 7 (Fig 2B). IL-4, IFN-γ and IL-17 were also tested but no significant induction was observed (data not shown). We then measured the induction of antimicrobial peptides, defensins (mBD3 and mBD14) and mouse cathelicidin (CRAMP), as important effectors of skin innate immunity [3,16]. PBre strain (EM) induced a significant amount of CRAMP with a peak at day 3 post-infection (Fig 2D). MR726 (MEM) strain strongly induced the defensin mBD-3 (Fig 2E). The wild-type strain 297 (CSF) exhibited a peak of mBD-3 at 24h post-infection while the 1808/03 strain (CSF) induced a negligible amount of all three AMPs tested (Fig 2D–2F).

### Specific analysis of the inflammation induced by *B. burgdorferi ss* 297 parental strain and its clone

**Fig 3:**
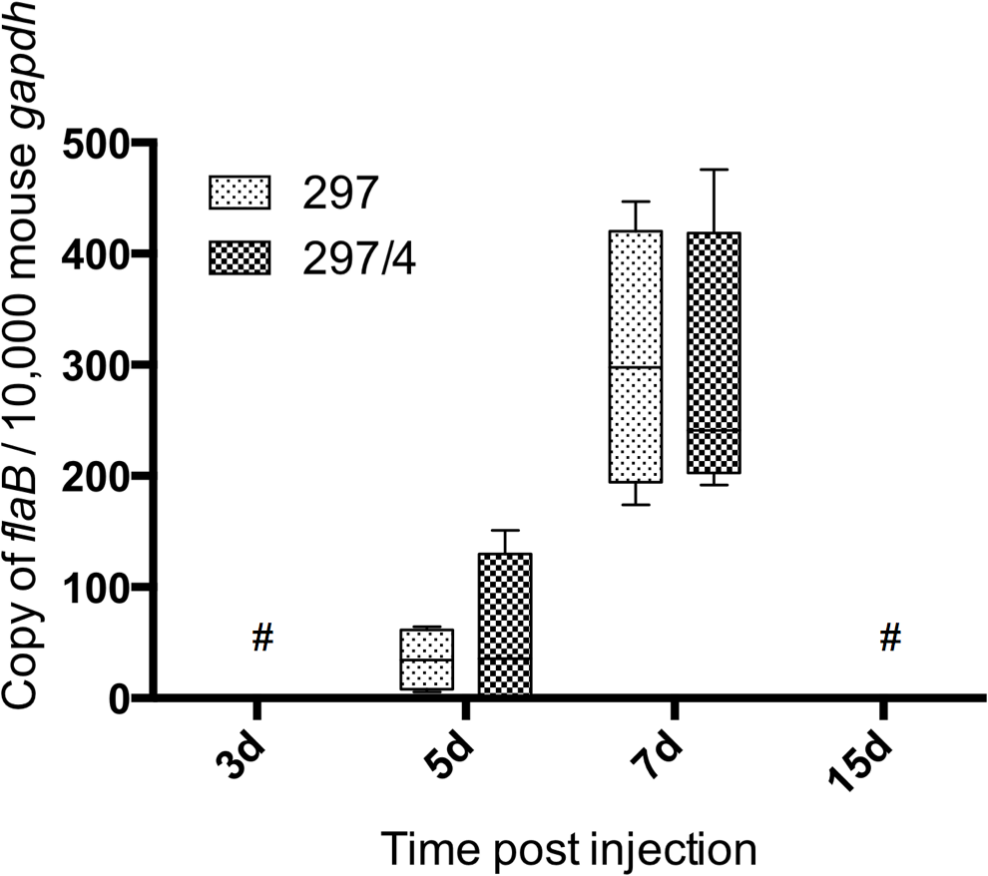
Bacterial loads in the skin at the inoculation site was measured by quantitative PCR for the *B*. *burgdorferi flaB* gene and normalized to copies of mouse *gapdh*. Values between strains were not statistically different (two-way ANOVA test using the Sidak-Bonferroni method). # values close to the detection limit.

Since *B*. *burgdorferi* infection might be triggered by a heterogeneous population in the vertebrate host, we performed the cloning of *B*. *burgdorferi* ss strain 297 [17]. We selected the clone 297/4 because of its cerebral dissemination at day 30 [18]. In addition, this clone induced neurological manifestations in a few mice (alteration of behavior and hemorraghes—data not shown). We compared the tissue distribution of the clone and the native strain: no significant difference was observed (Table 3). The quantification of *B*. *burgdorferi* load within the tissues confirmed the intense multiplication occurring in the skin at day 7 whatever the strain used, but no significant difference was observed between the clone and the parental population (Fig 3).

**Table 3:** Kinetics of tissue distribution of *Borrelia burgdorferi* 297 strain and its clone. Bacterial distribution was measured by culture (heart, skin at inoculation site, brain and ear) or by PCR (bladder and skin at the inoculation site) and by both methods for the ankle at different time points after intradermal injection of 10^3^ *B*. *burgdorferi*. Five mice were analyzed for each time point. h: hours; d: days; wt: wild type; c: clone.

	Tissue distribution											
	297 wt						c297/4					
	skin at inoculation site	heart	bladder	joint	ear	Brain	skin at inoculation site	heart	bladder	joint	ear	Brain
**24h**	**0/5**	**0/5**	**0/5**	**0/5**	**0/5**	**0/5**	**0/5**	**0/5**	**0/5**	**0/5**	**0/5**	**0/5**
**3d**	**5/5**	**1/5**	**0/5**	**0/5**	**0/5**	**0/5**	**4/5**	**1/5**	**0/5**	**0/5**	**0/5**	**0/5**
**5d**	**5/5**	**1/5**	**0/5**	**2/5**	**0/5**	**0/5**	**4/5**	**1/5**	**0/5**	**5/5**	**0/5**	**0/5**
**7d**	**5/5**	**4/5**	**5/5**	**5/5**	**2/5**	**1/5**	**4/5**	**4/5**	**5/5**	**5/5**	**0/5**	**1/5**
**15d**	**5/5**	**5/5**	**5/5**	**5/5**	**5/5**	**2/5**	**5/5**	**5/5**	**5/5**	**5/5**	**5/5**	**1/5**
**30d**	**5/5**	**5/5**	**5/5**	**5/5**	**5/5**	**2/5**	**5/5**	**5/5**	**5/5**	**5/5**	**5/5**	**1/5**

**Fig 4:**
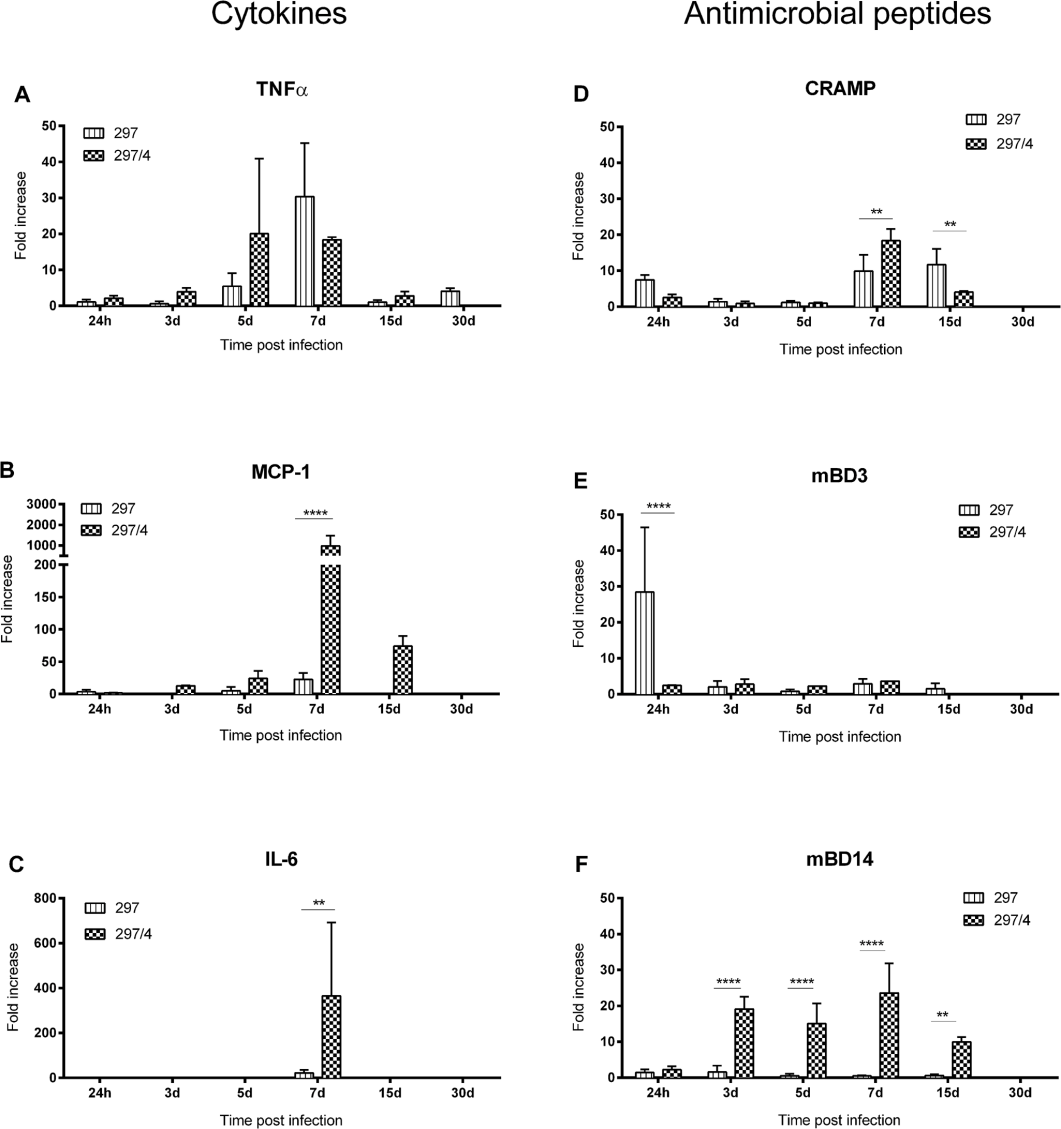
Inflammatory profiles of *B*. *burgdorferi* ss, parental strain and its clone 297/4. Levels of transcripts were measured by RT-qPCR from the skin at the inoculation site. The values were calculated using the 2-delta delta Ct method after normalization with *gapdh*. (h: hours, d: days). Two-way ANOVA was used to analyze the data. At least, three mice were analyzed for each time point.

Interestingly, when we compared the inflammatory profiles of the clone and the parental strain, clone 297/4 elicited a stronger skin inflammation with a high level of induction of MCP-1 and IL-6 (Fig 4). Similarly for the AMPs, we observed a significantly higher induction of defensin, mBD-14 and CRAMP for clone 297/4 compared to the parental strain (Fig 4D and 4F).

### Proteomic characterization of *B. burgdorferi ss*, 297, parental strain versus clone

**Fig 5:**
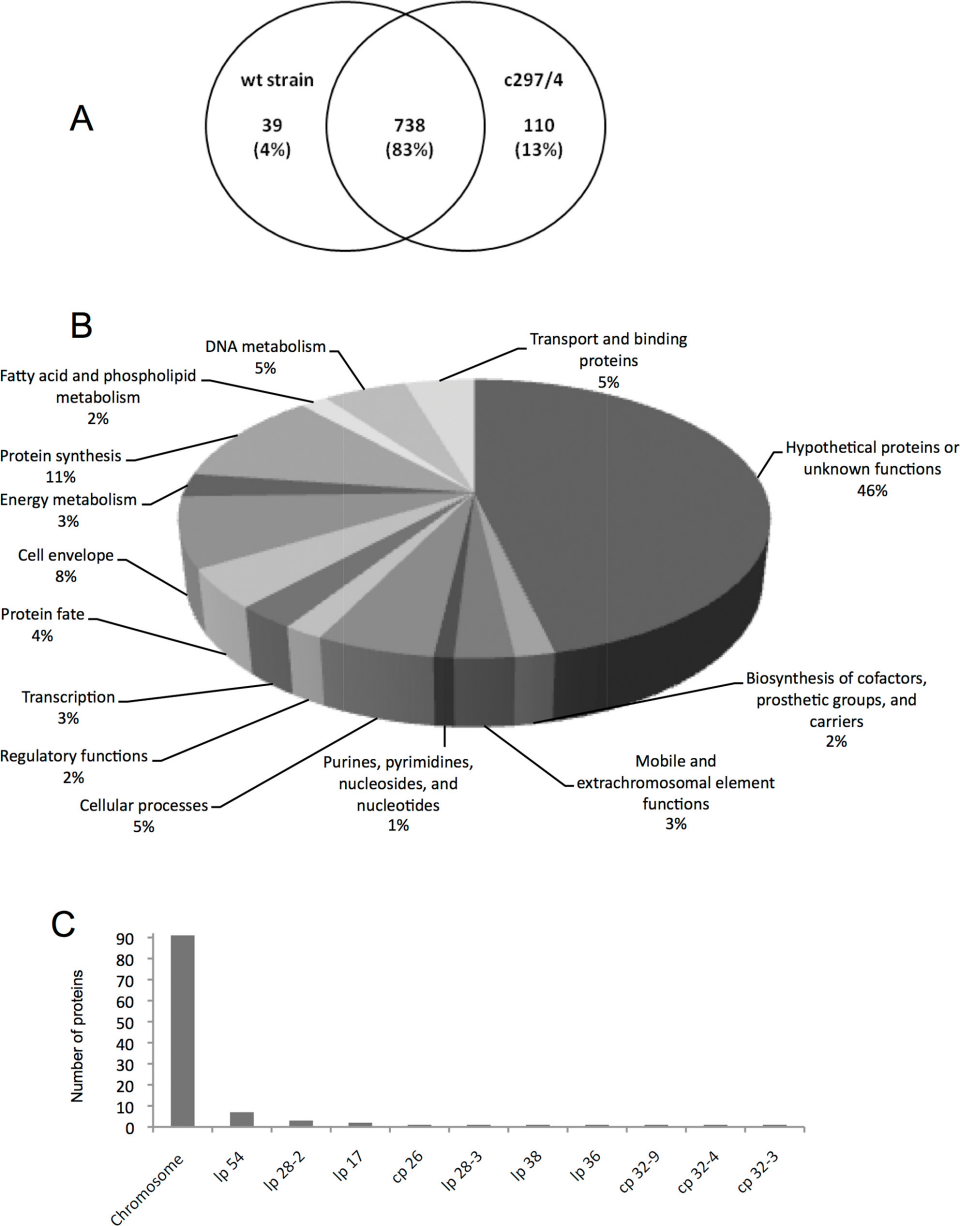
Proteomic analyses of *B*. *burgdorferi* ss, 297 parental strain and its clone 297/4. (A) The Venn diagram shows the protein overlap and the proteins specifically identified in the parental strain and the clone 297/4. (B) Graphic representation of the breakdown of the proteins specifically identified in the clone. Categorization was based upon JCVI annotation. The percentages represent the fraction of that category within the proteins. (C) Gene location of the specific proteins identified in the clone 297/4.

To determine whether a differential protein expression might explain the skin inflammatory profile triggered by the clone 297/4, we undertook a global proteomic analysis to compare the protein profiles of *B*. *burgdorferi* 297 and the clone 297/4. We used a GeLC-MS/MS based on SDS-PAGE, systematic excision of the protein bands, and identification through nanoLC-MS/MS as described [18]. This analytical strategy does not specifically target membrane proteins but affords an exhaustive identification of *B*. *burgdorferi* proteins. A total of 887 proteins were identified with 848 proteins in 297/4 and 777 proteins in the parental strain (Fig 5A and S2 Table). As compared to our previous results [18], we observed a reproducibility of 89% of the total proteins identified. Interestingly, in this study, 110 proteins were detected specifically in the clone 297/4. The breakdown of the biological functions of these proteins shows a high number of hypothetical or unknown protein functions (Fig 5B). The genome of *B*. *burgdorferi* B31 contains a single chromosome, 9 circular plasmids, and 12 linear plasmids. Among these 110 proteins, 91 are localized on the chromosome, 15 on linear plasmids, and 4 on circular plasmids (Fig 5C). However some plasmids present in *B*. *burgdorferi* B31 are absent in *B*. *burgdorferi* 297 (ie, 28–2). Several proteins (39) were detected only in the 297 parental strain and their under-expression in 297/4 could also contribute to bacterial specificity and heterogeneity (Fig 5A).

### Comparative induction of specific genes of *B. burgdorferi* 297 clone versus the parental strain in mouse skin by RT-PCR

**Fig 6:**
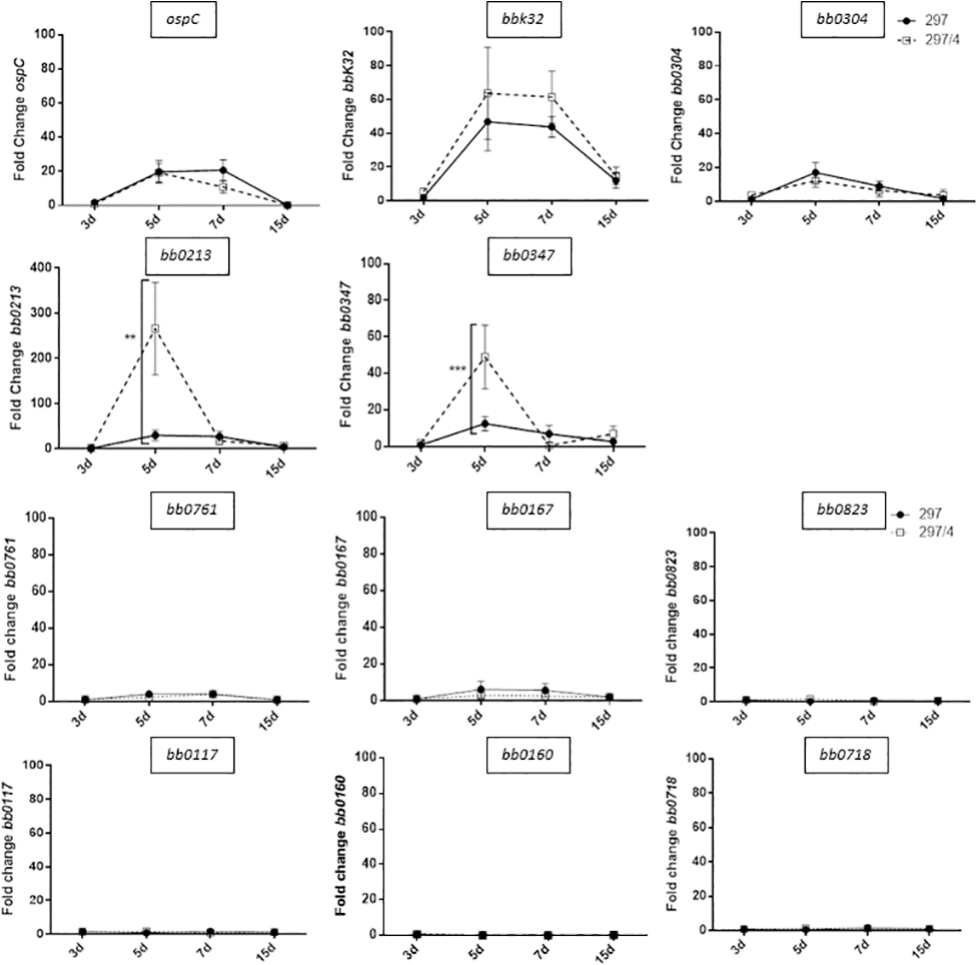
Measure of mRNA levels of *ospC*, *bbk32* genes as reference genes and 9 genes corresponding to cell envelope proteins, in the skin of mice infected with *B*. *burgdorferi* 297 parental strain or its clone 297/4 at different days after the bacterial inoculation. Two-way ANOVA was used to analyze the data. At least five mice were tested for each time point tested.

Since surface proteins of *B*. *burgdorferi* also represent candidates for bacterial diversity and are essential in the interaction with the host, we selected among the 110 specific proteins of the clone, those assigned to cell envelopes (S2 Table). By RT-PCR, we followed their gene expression during the skin inflammation in C3H/HeN mice. Among the 9 selected proteins, the level of mRNA of three of these proteins was significantly induced but without differences in the two bacterial populations: the two well-known genes, *ospC* and *bbk32* and *bb0304*, an enzyme involved in cell wall synthesis (Fig 6). Two genes were highly induced with a significant peak of expression at day 5 for the clone 297/4: *bb0213* and *bb0347*. All the other genes tested, *bb0761*, *bb0167*, *bb0160*, *bb0823*, *bb0117* and *bb0718* were weakly induced.

## Discussion

Considering that the skin of the vertebrate host is the first physical and immune barrier encountered by the spirochetes, it constitutes an efficient filter for arthropod-transmitted pathogens. The resident skin cells, keratinocytes and fibroblasts, play a pivotal role by recognizing pathogens via their Toll-like receptors. They also secrete anti-inflammatory molecules such as AMPs and chemokines [19–21]. Recently, the role of the skin has been re-explored with newly developed techniques and the skin now appears to be a critical step in the development of certain arthropod-borne diseases, such as leishmaniasis and malaria [22]. The biodiversity of *B*. *burgdorferi* ss varies according to its environment: it is greater in tick populations, less diverse in the skin, and even less diverse in disseminated sites such as the cerebrospinal fluid of patients [7]. The skin appears to constitute an interface, where among the very heterogeneous population of *Borrelia* in ticks, only specific OspC alleles are selected to disseminate in patients [8].

To further investigate the role of the skin barrier in Lyme borreliosis, we chose the mouse model [2,9] to study the outcome of various human clinical isolates that are responsible for different pathotypes. We investigated whether a specific inflammatory profile in the skin could be characteristic of a pathotype and could explain its organotropism. Because AMPs and other well-known pro-inflammatory molecules are important molecules of skin innate immunity [3,16] we studied their induction. AMPs are not only antimicrobial molecules they also stimulate chemokine production, angiogenesis, and wound healing. We have shown previously [15] that the *B*. *burgdorferi* ss strain N40 induces AMPs in the skin, especially the murine cathelicidin CRAMP, important in cutaneous inflammation [16,23]. The study of AMP induction in mouse skin after infection with different *B*. *burgdorferi* strains showed an induction of CRAMP in the presence of the PBre strain, while MR726 and 297 strains induced the defensin mBD-3 (an ortholog of human defensin hBD-2). With the human pathotype MR726 responsible for MEM, the mouse exhibited an important perturbation of its skin homeostasis.

We also characterized the RST genotype (data not shown) to obtain more complete information on the potential pathogenicity of the different *Borrelia* strains in the vertebrate host. As with the *ospC* gene, the *RST* genotype is defined as a disseminating marker. *B*. *burgdorferi* strains can be characterized by their genotype by RFLP as described previously, and this classification determines their invasiveness [9–12]. We investigated whether a specific RST could be associated with a specific inflammatory profile in the skin. PBre and 1808/03 strains, responsible for EM and neuroborreliosis in human respectively, were identified as being RST1, and the MR726 strain isolated from MEM lesions as RST3. RST1 is reported as a disseminating genotype while RST3 is considered as a non-invasive genotype [24]. In mice experimentally infected by different RSTs of *B*. *burgdorferi* ss, a clear difference was reported between RST1 and RST3 isolates. RST1 produces more severe symptoms and a heavier load of spirochete in different organs [25]. In our model, after syringe inoculation of *Borrelia*, the transmission did not differ significantly between PBre and 1808/03 (RST1) and MR726 (RST3) since the skin at the site of inoculation was positive at day 3 in both cases. The quantification in the skin did not differ either between these two RSTs. However, the inflammatory profile in the skin for the two RST1 (PBre and 1808/03) strains was slightly different with an induction of the cathelicidin CRAMP at day 3 for PBre and TNF-α at day 7 for both strains. The RST3 (MR726) strain tested, isolated from MEM, gave a strong inflammatory profile in the skin with a strong induction of the defensin mBD-3 and MCP-1. Overall, we showed a clear difference in skin innate immunity according to the bacterial strain tested, although we cannot correlate a human pathotype and an RST to a specific skin immune response in the mouse. In this sense, markers such as RST and OspC are not sufficient to determine the outcome of an infection initiated by a specific *Borrelia* strain and species due to the heterogeneity and biodiversity existing in *Borrelia* as shown in two recent studies [7,13].

Since the *Borrelia* population is heterogeneous, we decided to generate different clones from a well-characterized strain. We selected the strain *B*. *burgdorferi* 297, since this strain is particularly disseminating and is defined as RST2 (intermediate in dissemination). We chose clone 4 for its cerebral localization compared to the others we isolated [18]. A recent study in horses also revealed the cerebral localization of a strain of *B*. *burgdorferi* having 99,9% homology with *B*. *burgdorferi* ss 297 [26]. By its cerebral localization, this strain presents some interesting features of virulence. The clone and the parental strain both defined as RST2, multiplied similarly in the skin at day 7. However, the inflammatory profile induced by the clone 297/4 was significantly different from the profile observed with the parental strain, although the starting inoculum was identical for both strains. The clone 297/4 induces the defensin mBD-14 (an ortholog of human defensin 3 or hBD3) and more importantly of MCP-1 and IL-6. AMPs are chemoattractant for immune cells and also possess angiogenic properties [3] that could be used by pathogens to better diffuse via the blood to target organs. Recently, mBD-14 has been shown to promote angiogenesis [27]. Similarly, the induction of MCP-1 by the clone could also facilitate the diffusion of the bacteria in the host tissues, since it has been described to induce the permeability of vascular endothelium cells in dengue hemorrhagic fever [28]. Another recent study by Rego et al. [13] confirms our observation concerning the heterogeneity of *Borrelia* populations. They showed that clones behave and persist differently *in vivo*. They generated clones from the parental strain *B*. *burgdorferi* ss B31. Each clone was distinguishable by a specific sequence tag. They simultaneously inoculated 7 clones and then conducted a follow up study. Stochastic selection occurred at the different steps of *Borrelia* acquisition and transmission. Interestingly, 9 weeks after the inoculation, not all mice were infected with a specific clone and some clones persisted better than others.

Since surface proteins could be specifically involved in the process of dissemination, persistence and organotropism of *Borrelia*, we carried out a global proteomic analysis to compare these two populations. Up to now proteomic approaches, using electrophoresis and mass spectrometry have been described mainly to investigate the immunoreactive *Borrelia* proteins [29–31]. Only few global proteomic approaches have been described for the analysis of protein profiles in *Borrelia* [32–34]. The detection of 110 proteins only in the clone 297/4 can be explained by an under-expression in the parental strain and/or an over-expression in the clone. Either the 297 parental strain does not express these proteins, or more likely their expression level is below the limit of detection by GeLC-MS/MS. We then focused on the relative expression of 9 selected surface protein genes *in vivo*, since such proteins have been described as particularly important in the infectivity and potentially in *Borrelia* persistence and dissemination. In mouse skin, the two well-known genes, *ospC* and *bbk32* were both well induced by the two populations but no particular profile was observed. Similar profile was observed for *bb0304*, a murF enzyme that catalyzes the murein synthesis involved in bacterial cell wall formation. In contrast, the clone upregulated two genes at day 5, *bb0347*, related to a fibronectin binding protein and *bb0213*, a hypothetical protein. It is well established that the interaction of *Borrelia* with the extracellular matrix is essential for its dissemination and persistence [35–39]. In addition, *bb0347* has recently been described as a ligand for the human fibronectin [38]. The behavior of the clone seems to be correlated to an overall higher gene induction at day 5, especially for three surface-exposed proteins.

The skin by its immunity and the specificity of its different resident cells likely plays a major role in the development of *B*. *burgdorferi* infection in the vertebrate host. There, an intense bacterial multiplication occurs. Our study demonstrates that the skin is a privileged site for *B*. *burgdorferi*, and the development of cutaneous immunity might be crucial for the adaptation of *B*. *burgdorferi* in the vertebrate host. We showed that RST genetic markers are not sufficient to define the outcome of *B*. *burgdorferi* in the host. *Borrelia* inoculum is heterogeneous and the bacterium expresses essential factors for its dissemination and persistence that are not solely linked to the genetic markers *ospC* and RST, as shown by the data presented here and others [7,13] Some specific factors of both, the bacteria (eg OspC, BB0347 and other unknown proteins) and the host (eg AMPs and MCP-1), display a sophisticated interaction that likely further orients the bacterium to specific organs of the vertebrate host. All these specific factors remain to be identified to understand more clearly the physiopathology of the disease that is initiated in the skin.

## Materials and Methods

### Mouse and bacterial strains

Three to four week old C3H/HeN pathogen-free mice were purchased from Charles River Laboratories (L'Arbresle, France) and provided food and water *ad libitum*.

Bacterial isolates belonging to *Borrelia burgdorferi* sensu stricto species were recovered from patients with different clinical manifestations and therefore defined as pathotypes. PBre strain was isolated from a unique *erythema migrans* lesion (EM) in Germany, MR726 strain from a multiple *erythema migrans* (MEM) lesion in the United States, 1808/03 strain from cerebrospinal fluid in Slovenia and 297 strain from cerebrospinal fluid in the United States. The *B*. *burgdorferi* ss clones were obtained by culture on solid BSK medium [17]. For its dissemination profile, the clone 297/4 was used in our comparative study with its native strain [18].

All the strains were cultured in BSK-H complete medium (Sigma) at 33°C and used at low passage (<7) for mouse infection. Spirochetes were counted and viability was checked using dark field microscopy.

### Follow up of mouse infection and tissue sampling

Mice were infected with 10^3^ spirochetes in 0.1 mL BSK by intradermal injection in the dorsal thoracic area. Serology was performed as previously described [40]. At different time points after the beginning of the experiment (0h, 24h, 3d, 5d, 7d, 15d and 30d post-infection), mice were killed by isoflurane gas overdose. About a 1 cm area of mouse skin was collected at the site of the inoculation and stored in Trizol reagent (Invitrogen). The ear, the heart, the bladder and the ankle from each mouse were harvested aseptically and each organ was divided into 2 pieces, for PCR and culture in BSK-H medium. Organs of an uninfected mouse were simultaneously collected as a negative control.

### Detection of *B. burgdorferi* by culture and by PCR in mouse organs

Collected organs were placed in 6 ml of BSK-H medium containing 30μg of rifampicin (BioRad). The tubes were maintained at 33°C and examined weekly for the presence of spirochetes by dark-field microscopy as described previously [15].

For PCR detection, DNA was extracted from the organs of individual mice on a MagNA Pure system (Roche Diagnostics, France) using a MagNA Pure LC large-volume DNA isolation kit after external lysis. Briefly, heart, urinary bladder, ear and skin were added to 500μL of lysis buffer containing proteinase K. Ankle specimens were treated with an external lysis by collagenase A and then proteinase K. All DNA samples were finally eluted into 100 μL of elution buffer. Ten μL of eluted DNA were used as a template for *B*. *burgdorferi* detection. Qualitative amplification, targeting the *flagellin* gene, was carried out as described [40].

### Quantification of spirochete load and measure of inflammatory gene induction in mouse skin by RT-PCR

At the site of inoculation, quantification of the *B*. *burgdorferi* ss-specific *flagellin* gene was carried out on a LightCycler system (Roche Diagnostics, France). The primers used to amplify the *fla* gene were those previously described [15].

To measure the inflammation at the inoculation site, total RNA was extracted from 10 mg of mouse skin using Trizol Reagent as recommended by the manufacturer (Invitrogen). Samples were treated by DNAse (Ambion, USA). Then first-strand cDNA was synthesized from 1 μg of total RNA using SuperScript II reverse transcriptase (Invitrogen Life Technologies). *gapdh* quantification was performed as an internal standard. Relative expression levels were calculated using cDNAs from three uninfected mice as a calibrator. Amplification and detection were performed with an ABI 7000 system with the following thermal profile: 95°C for 10 min, 50 cycles of 95°C for 15 s, 60°C for 1 min. Primers for all the genes studied are listed in S1 Table or have already been described [15].

### Comparison of the protein profile of *B. burgdorferi* strain 297, wild-type and clone, by protein fractionation, in gel digestion and nanoLC-MS/MS

Cultures of *B*. *burgdorferi* 297, parental strain and clone 297/4, were analysed as previously described [18], using 12% SDS-PAGE and a nanoLC-Chip/MS (Agilent Technologies, Palo Alto, CA) hyphenated to an ion trap amaZon (BrukerDaltonics, Bremen, Germany). Mass data were interpreted using the Mascot 2.3.02. (Matrix Science, London, UK) and the OMSSA 2.1.7 (Open Mass Spectrometry Search Algorithm, Maryland, USA) algorithms. Searches were performed against an in house generated protein database composed of protein sequences of *B*. *burgdorferi* ss B31 and known contaminant proteins such as human keratins and trypsin, downloaded from NCBI non redundant database concatenated with reversed copies of all sequences (total 2002 entries). This database was used because the *B*. *burgdorferi* ss 297 strain has not yet been completely sequenced. The Mascot and OMSSA results were independently loaded into the Scaffold software (Proteome Software, Portland, OR). The target-decoy database search allowed us to control the false positive identification rate which was set to 1%.

### Dynamics of gene expression of surface proteins of *B. burgdorferi* 297, parental strain and clone 297/4, in mouse skin at the inoculation site

At different time points, skin samples were collected from each mouse at the inoculation site. Total RNA was purified using Trizol reagent according to the manufacturer’s instructions. The concentration and purity of extracted RNA were determined by measuring the A260 and A280. Samples were then treated with gDNAse wipeout (QIAGEN) before testing for DNA contamination. The total extracted RNA was subjected to synthesize cDNA using Quantiscript Reverse Transcription (QIAGEN). cDNA was used to quantify the well-known genes *ospC* and *bbk32* levels as positive controls. For *B*. *burgdorferi* ss 297/4, genes corresponding to cell envelope proteins (S2 Table) were selected for the RT-PCR. Relative expression levels were calculated using the ∆∆Ct method with *flagellin* as the internal standard. Amplification and detection were performed with an ABI 7500 system with the following thermal profile: 95°C for 10 min, 50 cycles of 95°C for 15 s, 50°C for 30 s and 60°C for 1 min. Each amplification condition was compared to that of the day 3 for relative quantification. Correlation factors were calculated by comparing cDNA amplification of the native strain to cDNA amplification of the clone for each day. Then, the curve obtained for the clone was normalized by these factors to get a second curve, representative of the native strain, and quantitatively comparable to the clone.

### Ethics Statement

The protocols carried out in this study were in accordance and approved by the CREMEAS Committee on the Ethics of Animal Experiments of the University of Strasbourg (Comité Régional d’Ethique en Matière d’Expérimentation Animale Strasbourg—Permit Number: AI/10/39/12/12).

### Statistical analyses

For the different kinetics study, at least two mouse cohorts were used to get at least 5 mice for each time point. For qRT-PCR, at least two to three extractions were made by mouse skin biopsies. For the qRT-PCR results, gene expression relative to control is reported. Error bars represent the SD from at least three independent experiments and each time point of the kinetic corresponds to 2 to 3 mice. The statistical significance of differences was determined using the two-way ANOVA test. A p-value of 0.05 was considered statistically significant. All statistical analyses were performed with Prism 6 software (Graphpad, La Jolla, CA).

## Supporting Information

S1 Table Primers designed for this study. (DOCX)

S2 Table Proteins specifically detected in *B*. *burgdorferi* ss, clone c297/4. The 110 proteins identified specifically in the clone are given with their gene location and biological function from JCVI annotation. ^1^ Represents proteins detected by the search engine OMSSA. ^2^ Represents proteins detected by the search engine Mascot. In bold: proteins assigned to cell envelop.
(DOCX)

## References

[1] Stanek G, Wormser GP, Gray J, Strle F. Lyme borreliosis. Lancet. 2012;379: 461–473. 10.1016/S0140-6736(11)60103-7 21903253

[2] Barthold SW, de Souza MS, Janotka JL, Smith AL, Persing DH. Chronic Lyme borreliosis in the laboratory mouse. Am. J. Pathol. 1993;143: 959–971. 8362988 PMC1887206

[3] Gallo RL, Hooper LV. Epithelial antimicrobial defence of the skin and intestine. Nature. 2012;12: 503–516. 10.1038/nri3228 PMC3563335 22728527

[4] Hodzic E, Feng S, Freet KJ, Borjesson DL, Barthold SW. *Borrelia burgdorferi* population kinetics and selected gene expression at the host-vector interface. Infect. Immun. 2002;70: 3382–3388. 12065476 10.1128/IAI.70.7.3382-3388.2002 PMC128091

[5] Antonara S, Ristow L, McCarthy J, Coburn J. Effect of *Borrelia burgdorferi* OspC at the site of inoculation in mouse skin. Infect. Immun. 2010;78: 4723–4733. 10.1128/IAI.00464-10 20696825 PMC2976318

[6] Tilly K, Bestor A, Jewett MW, Rosa P. Rapid clearance of Lyme disease spirochetes lacking OspC from skin. Infect. Immun. 2007;75: 1517–9. 17158906 10.1128/IAI.01725-06 PMC1828573

[7] Brisson D, Baxamusa N, Schwartz I, Wormser GP. Biodiversity of *Borrelia burgdorferi* strains in tissues of Lyme disease patients. PLoS ONE 2011;6:e22926 10.1371/journal.pone.0022926 21829670 PMC3150399

[8] Baranton G, De Martino SJ. *Borrelia burgdorferi* sensu lato diversity and its influence on pathogenicity in humans. Curr. Probl. Dermatol. 2009;37: 1–17. 10.1159/000213066 19367094

[9] Liveris D, Wormser GP, Nowakowski J, Nadelman RB, Bittker S, Cooper D, et al Molecular typing of *Borrelia burgdorferi* from Lyme disease patients by PCR-restriction fragment length polymorphism analysis. J. Clin. Microbiol. 1996;34: 1306–1309. 8727927 10.1128/jcm.34.5.1306-1309.1996 PMC229006

[10] Seinost G, Dykhuizen DE, Dattwyler RJ, Golde WT, Dunn JJ, Wang IN, et al Four clones of *Borrelia burgdorferi* sensu stricto cause invasive infection in humans. Infect. Immun. 1999;67: 3518–3524. 10377134 10.1128/iai.67.7.3518-3524.1999 PMC116539

[11] Wormser GP, Brisson D, Liveris D, Hanincová K, Sandigursky S, Nowakowski J, et al *Borrelia burgdorferi* Genotype Predicts the Capacity for Hematogenous Dissemination during Early Lyme Disease. J Infect Dis. 2008;198: 1358–1364. 10.1086/592279 18781866 PMC2776734

[12] Jones KL, Glickstein LJ, Damle N, Sikand VK, McHugh GA, Steere AC. *Borrelia burgdorferi* Genetic Markers and Disseminated Disease in Patients with Early Lyme Disease. J. Clin. Microbiol. 2006;44: 4407–13. 17035489 10.1128/JCM.01077-06 PMC1698394

[13] Rego ROM, Bestor A, Stefka J, Rosa PA. Population bottlenecks during the infectious cycle of the Lyme disease spirochete *Borrelia burgdorferi* . PLoS ONE 2014;9:e101009 10.1371/journal.pone.0101009 24979342 PMC4076273

[14] Hodzic E, Feng S, Freet KJ, Barthold SW. *Borrelia burgdorferi* population dynamics and prototype gene expression during infection of immunocompetent and immunodeficient mice. Infect. Immun. 2003;71: 5042–5055. 12933847 10.1128/IAI.71.9.5042-5055.2003 PMC187352

[15] Kern A, Collin E, Barthel C, Michel C, Jaulhac B, Boulanger N. Tick Saliva Represses Innate Immunity and Cutaneous Inflammation in a Murine Model of Lyme Disease. Vector-Borne Zoonotic Dis. 2011;11: 1343–1350. 10.1089/vbz.2010.0197 21612525

[16] Schauber J, Gallo RL. Antimicrobial peptides and the skin immune defense system. J. Allergy Clin. Immunol. 2009;124: R13–8. 10.1016/j.jaci.2009.07.014 19720207

[17] De Martino SJ, Sordet C, Piémont Y, Ruzic-Sabljic E, Thaddée Vetter M, Monteil H, et al Enhanced culture of *Borrelia garinii* and *Borrelia afzelii* strains on a solid BSK-based medium in anaerobic conditions. Res. Microbiol. 2006;157: 726–729. 16814991 10.1016/j.resmic.2006.05.002

[18] Schnell G, Boeuf A, Jaulhac B, Boulanger N, Collin E, Barthel C, et al Proteomic analysis of three *Borrelia burgdorferi* sensu lato native species and disseminating clones: Relevance for Lyme vaccine design. Proteomics. 2015;15: 1280–90. 10.1002/pmic.201400177 25475896

[19] Nestle FO, Di Meglio P, Qin J-Z, Nickoloff BJ. Skin immune sentinels in health and disease. Nat. Rev. Immunol. 2009;9: 679–691. 10.1038/nri2622 19763149 PMC2947825

[20] Marchal CMP, Luft BJ, Yang X, Sibilia J, Jaulhac B, Boulanger NM. Defensin is suppressed by tick salivary gland extract during the *in vitro* interaction of resident skin cells with *Borrelia burgdorferi* . J. Invest. Dermatol. 2009;129: 2515–2517. 10.1038/jid.2009.73 19340008

[21] Marchal C, Schramm F, Kern A, Luft BJ, Yang X, Schuijt T, et al Antialarmin effect of tick saliva during the transmission of Lyme disease. Infect. Immun. 2011;79: 774–785. 10.1128/IAI.00482-10 21134970 PMC3028856

[22] Bernard Q, Jaulhac B, Boulanger N. Smuggling across the border: how arthropod-borne pathogens evade and exploit the host defense system of the skin. J. Invest. Dermatol. 2014;134: 1211–1219. 10.1038/jid.2014.36 24552683

[23] Nizet V, Ohtake T, Lauth X, Trowbridge J, Rudisill J, Dorschner RA, et al Innate antimicrobial peptide protects the skin from invasive bacterial infection. Nature. 2001;414: 454–457. 11719807 10.1038/35106587

[24] Wormser GP, Liveris D, Nowakowski J, Nadelman RB, Cavaliere LF, McKenna D, et al Association of specific subtypes of *Borrelia burgdorferi* with hematogenous dissemination in early Lyme disease. J Infect Dis. 1999;180: 720–725. 10438360 10.1086/314922

[25] Wang G, Ojaimi C, Iyer R, Saksenberg V, McClain SA, Wormser GP, et al Impact of genotypic variation of *Borrelia burgdorferi* sensu stricto on kinetics of dissemination and severity of disease in C3H/HeJ mice. Infect. Immun. 2001;69: 4303–4312. 11401967 10.1128/IAI.69.7.4303-4312.2001 PMC98500

[26] Imai DM, Barr BC, Daft B, Bertone JJ, Feng S, Hodzic E, et al Lyme neuroborreliosis in 2 horses. Vet. Pathol. 2011;48: 1151–1157. 10.1177/0300985811398246 21285382

[27] Röhrl J, Huber B, Koehl GE, Geissler EK, Hehlgans T. Mouse β-defensin 14 (Defb14) promotes tumor growth by inducing angiogenesis in a CCR6-dependent manner. J. Immunol. 2012;188:4931–4939. 10.4049/jimmunol.1102442 22504651

[28] Lee YR, Liu MT, Lei HY, Liu CC, Wu JM, Tung YC, et al MCP-1, a highly expressed chemokine in dengue haemorrhagic fever/dengue shock syndrome patients, may cause permeability change, possibly through reduced tight junctions of vascular endothelium cells. J. Gen. Virol. 2006;87: 3623–3630. 17098977 10.1099/vir.0.82093-0

[29] Jungblut PR, Grabher G, Stöffler G. Comprehensive detection of immunorelevant *Borrelia garinii* antigens by two-dimensional electrophoresis. Electrophoresis. 1999;20: 3611–3622. 10612288 10.1002/(SICI)1522-2683(19991201)20:18<3611::AID-ELPS3611>3.0.CO;2-4

[30] Nowalk AJ, Gilmore RD, Carroll JA. Serologic proteome analysis of *Borrelia burgdorferi* membrane-associated proteins. Infect. Immun. 2006;74: 3864–3873. 16790758 10.1128/IAI.00189-06 PMC1489744

[31] Nowalk AJ, Nolder C, Clifton DR, Carroll JA. Comparative proteome analysis of subcellular fractions from *Borrelia burgdorferi* by NEPHGE and IPG. Proteomics. 2006;6: 2121–2134. 16485259 10.1002/pmic.200500187

[32] Angel TE, Luft BJ, Yang X, Nicora CD, Camp DG, Jacobs JM, et al Proteome analysis of *Borrelia burgdorferi* response to environmental change. PLoS ONE. 2010;5:e13800 10.1371/journal.pone.0013800 21072190 PMC2970547

[33] Gesslbauer B, Poljak A, Handwerker C, Schüler W, Schwendenwein D, Weber C, et al Comparative membrane proteome analysis of three *Borrelia* species. Proteomics. 2012;12: 845–858. 10.1002/pmic.201100211 22539435

[34] Jacobs JM, Yang X, Luft BJ, Dunn JJ, Camp DG, Smith RD. Proteomic analysis of Lyme disease: global protein comparison of three strains of *Borrelia burgdorferi* . Proteomics. 2005;5: 1446–1453. 15800874 10.1002/pmic.200401052

[35] Pal U, Fikrig E. Adaptation of *Borrelia burgdorferi* in the vector and vertebrate host. Microbes Infect. 2003;5: 659–66. 12787742 10.1016/s1286-4579(03)00097-2

[36] Seshu J, Esteve-Gassent MD, Labandeira-Rey M, Kim JH, Trzeciakowski JP, Höök M, et al Inactivation of the fibronectin-binding adhesin gene bbk32 significantly attenuates the infectivity potential of *Borrelia burgdorferi* . Mol. Microbiol. 2006;59: 1591–1601. 16468997 10.1111/j.1365-2958.2005.05042.x

[37] Russell TM, Delorey MJ, Johnson BJB. *Borrelia burgdorferi*/BbHtrA degrades host ECM proteins and stimulates release of inflammatory cytokines in vitro. Mol. Microbiol. 2013;90: 241–251. 10.1111/mmi.12377 23980719

[38] Gaultney RA, Gonzalez T, Floden AM, Brissette CA. BB0347, from the lyme disease spirochete *Borrelia burgdorferi*, is surface exposed and interacts with the CS1 heparin-binding domain of human fibronectin. PLoS ONE 2013;8:e75643 10.1371/journal.pone.0075643 24086600 PMC3785480

[39] Ellis TC, Jain S, Linowski AK, Rike K, Bestor A, Rosa PA, et al *In Vivo* Expression Technology Identifies a Novel Virulence Factor Critical for *Borrelia burgdorferi* Persistence in Mice. PLoS Pathog. 2013;9:e1003567 10.1371/journal.ppat.1003567 24009501 PMC3757035

[40] Woods A, Soulas-Sprauel P, Jaulhac B, Arditi B, Knapp A-M, Pasquali J-L, et al MyD88 negatively controls hypergammaglobulinemia with autoantibody production during bacterial infection. Infect. Immun. 2008;76: 1657–1667. 10.1128/IAI.00951-07 18227170 PMC2292895

