# Supplementary material for: Heterogeneity of Borrelia burgdorferi Sensu Stricto Population and Its Involvement in Borrelia Pathogenicity: Study on Murine Model with Specific Emphasis on the Skin Interface
Source: PLoS One. 2015 Jul 21;10(7):e0133195. doi: 10.1371/journal.pone.0133195 (PMC4510351; doi:10.1371/journal.pone.0133195)
Supplement: S1 Table — (DOCX) [file pone.0133195.s001.docx]

**Table S1**

| **Genes** | **Primers** |
| --- | --- |
| ***IL-6*** | F 5’-tagtccttcctaccccaatttcc-3’  R 5’-ttggtccttagccactccttc-3’ |
| ***bbk32*** | F 5’-CAACAAAGCTAACCCAAATGTAT-3’ R 5’-CTTTTGTAAACTTTGCAGCTTCT -3’ |
| ***ospC*** | F 5’-ATAATTCAGGAAAAGATGGGAAT-3’  R 5’-GCCAGAACAACTGCGTTAG-3’ |
| ***bb0117*** | F-5’ AGGCCCTCAGCGGAAAAGGA 3’  R-5’ GCCAAAAGTGAGATGCTGATGCGA 3’ |
| ***bb0160*** | F-5’ ATGTATAATAATAAAACAATGG 3’  R-5’ ATTTTCTCTTTTCGTATTTTCC 3’ |
| ***bb0167*** | F-5’ CCAAGATTCCCAAAAACACC 3’  R-5’ TAATGCTTGCCATTGTGCTC 3’ |
| ***bb0213*** | F-5’ TTTGCTTGTTCTTGCGACAT 3’  R-5’ TCCTTTCCAAGGCTAGAATCA 3’ |
| ***bb0304*** | F-5’ AGCAAAAATCGCAAATCCAA 3’  R-5’ ATATTATGCCGACCCAGCAA 3’ |
| ***bb0347*** | F-5’ ACCAAAAGAAAATGCCTTGC 3’  R-5’ CAAGCCTATTTTTGGCGTTT 3’ |
| ***bb0718*** | F-5’ TCTGTTTACCCGCCTGCGTCT 3’  R-5’ TGACCTCCGGGCTTCCAGCA 3’ |
| ***bb0761*** | F-5’ AAATGGATGGCAGTCCGGCATT 3’  R-5’ CTCTTGCCCAACACTCACAGCAGG 3’ |
| ***bb0823*** | F-5’ CAGCATTCTTGGCTTTAGCA 3’  R-5’ CAATTCCATAGCGCTCTGTTT 3’ |
