# Supplementary material for: Heterogeneity of Borrelia burgdorferi Sensu Stricto Population and Its Involvement in Borrelia Pathogenicity: Study on Murine Model with Specific Emphasis on the Skin Interface
Source: PLoS One. 2015 Jul 21;10(7):e0133195. doi: 10.1371/journal.pone.0133195 (PMC4510351; doi:10.1371/journal.pone.0133195)
Supplement: S2 Table — The 110 proteins identified specifically in the clone are given with their gene location and biological function from JCVI annotation. 1 Represents proteins detected by the search engine OMSSA. 2 Represents proteins detected by the search engine Mascot. In bold: proteins assigned to cell envelop. (DOCX) [file pone.0133195.s002.docx]

**Table S2.**

| **Protein name** | **Locus tag** | **Biological function** | **Localization** |
| --- | --- | --- | --- |
| hypothetical protein BB0001^1^ | BB0001 | Hypothetical proteins | Chromosome |
| phosphoglucomutase (femD)^1,2^ | BB0004 | Energy metabolism | Chromosome |
| tryptophanyl-tRNA synthetase^1,2^ | BB0005 | Protein synthesis | Chromosome |
| Holliday junction DNA helicase RuvB^1,2^ | BB0022 | DNA metabolism | Chromosome |
| hypothetical protein BB0029^1,2^ | BB0029 | Unknown function | Chromosome |
| 1-acyl-sn-glycerol-3-phosphate acetyltransferase (plsC)^1,2^ | BB0037 | Fatty acid and phospholipid metabolism | Chromosome |
| hypothetical protein BB0043^1,2^ | BB0043 | Hypothetical proteins | Chromosome |
| ribonuclease HII^1,2^ | BB0046 | Transcription | Chromosome |
| hypothetical protein BB0049^1,2^ | BB0049 | Hypothetical proteins | Chromosome |
| hypothetical protein BB0058^1,2^ | BB0058 | Unknown function | Chromosome |
| peptide deformylase^1,2^ | BB0065 | Protein fate | Chromosome |
| hypothetical protein BB0066^1,2^ | BB0066 | Hypothetical proteins | Chromosome |
| hypothetical protein BB0097^1,2^ | BB0097 | Hypothetical proteins | Chromosome |
| hypothetical protein BB0102^1,2^ | BB0102 | Hypothetical proteins | Chromosome |
| **hemolysin III (yplQ)^2^** | **BB0117** | **Cell envelope** | **Chromosome** |
| hypothetical protein BB0156^1,2^ | BB0156 | Hypothetical proteins | Chromosome |
| **alanine racemase (alr)^1^** | **BB0160** | **Cell envelope** | **Chromosome** |
| hypothetical protein BB0161^1^ | BB0161 | Hypothetical proteins | Chromosome |
| hypothetical protein BB0165^1,2^ | BB0165 | Hypothetical proteins | Chromosome |
| **outer membrane protein (tpn50)^1^** | **BB0167** | **Cell envelope** | **Chromosome** |
| hypothetical protein BB0170^1^ | BB0170 | Hypothetical proteins | Chromosome |
| hypothetical protein BB0192^1^ | BB0192 | Hypothetical proteins | Chromosome |
| protoporphyrinogen oxidase, putative^1^ | BB0197 | Biosynthesis of cofactors, prosthetic groups, and carriers | Chromosome |
| hemolysin, putative^1,2^ | BB0202 | Cellular processes | Chromosome |
| **hypothetical protein BB0213^1,2^** | **BB0213** | **Cell envelope** | **Chromosome** |
| phosphate ABC transporter, permease protein (pstC)^1,2^ | BB0216 | Transport and binding proteins | Chromosome |
| flagellar motor switch protein (fliG-1)^1,2^ | BB0221 | Cellular processes | Chromosome |
| ribosomal protein S20 (rpsT)^2^ | BB0233 | Protein synthesis | Chromosome |
| hypothetical protein BB0244^1,2^ | BB0244 | Hypothetical proteins | Chromosome |
| cell division protein, putative^1,2^ | BB0257 | Protein fate | Chromosome |
| signal peptidase I (lepB-3)^1^ | BB0263 | Protein fate | Chromosome |
| flagellar biosynthesis protein FliP^1,2^ | BB0275 | Cellular processes | Chromosome |
| flagellar protein (flbC)^1^ | BB0285 | Cellular processes | Chromosome |
| **UDP-N-acetylmuramoylalanyl-D-glutamyl-2,6-diaminopimelate--D-alanyl-D-alanine ligase (murF)^1,2^** | **BB0304** | **Cell envelope** | **Chromosome** |
| hypothetical protein BB0308^1,2^ | BB0308 | Hypothetical proteins | Chromosome |
| hypothetical protein BB0322^1,2^ | BB0322 | Hypothetical proteins | Chromosome |
| **fibronectin/fibrinogen-binding protein, putative^2^** | **BB0347** | **Cell envelope** | **Chromosome** |
| hypothetical protein BB0349^1,2^ | BB0349 | Hypothetical proteins | Chromosome |
| 16S ribosomal RNA methyltransferase RsmE^1^ | BB0358 | Protein synthesis | Chromosome |
| hypothetical protein BB0409^1,2^ | BB0409 | Hypothetical proteins | Chromosome |
| endonuclease precursor (nucA)^1,2^ | BB0411 | Unknown function | Chromosome |
| 3-methyladenine DNA glycosylase (mag)^1,2^ | BB0422 | DNA metabolism | Chromosome |
| hypothetical protein BB0432^1,2^ | BB0432 | Hypothetical proteins | Chromosome |
| hypothetical protein BB0439^1,2^ | BB0439 | Hypothetical proteins | Chromosome |
| Na+/H+ antiporter (napA)^1,2^ | BB0447 | Transport and binding proteins | Chromosome |
| hypothetical protein BB0453^1^ | BB0453 | Transport and binding proteins | Chromosome |
| hypothetical protein BB0455^1,2^ | BB0455 | Transcription | Chromosome |
| hypothetical protein BB0510^1,2^ | BB0510 | Hypothetical proteins | Chromosome |
| hypothetical protein BB0526^1,2^ | BB0526 | Hypothetical proteins | Chromosome |
| pantothenate kinase^1^ | BB0527 | Regulatory functions | Chromosome |
| xylulokinase (xylB)^1,2^ | BB0545 | Energy metabolism | Chromosome |
| hypothetical protein BB0554^1,2^ | BB0554 | Hypothetical proteins | Chromosome |
| hypothetical protein BB0576^1,2^ | BB0576 | Hypothetical proteins | Chromosome |
| dimethyladenosine transferase^1^ | BB0590 | Protein synthesis | Chromosome |
| L-lactate permease (lctP)^1,2^ | BB0604 | Transport and binding proteins | Chromosome |
| rep helicase, single-stranded DNA-dependent ATPase (rep)^1,2^ | BB0607 | DNA metabolism | Chromosome |
| hypothetical protein BB0626^1,2^ | BB0626 | Unknown function | Chromosome |
| exodeoxyribonuclease V, alpha chain (recD)^1,2^ | BB0632 | DNA metabolism | Chromosome |
| ribosomal biogenesis GTPase^1,2^ | BB0643 | Protein synthesis | Chromosome |
| serine/threonine kinase, putative^1,2^ | BB0648 | Protein fate | Chromosome |
| GTP-binding protein Era^1,2^ | BB0660 | Protein synthesis | Chromosome |
| ribose/galactose ABC transporter, permease protein (rbsC-1)^1,2^ | BB0678 | Transport and binding proteins | Chromosome |
| tRNA-specific 2-thiouridylase MnmA^1,2^ | BB0682 | Protein synthesis | Chromosome |
| tRNA (guanine-N(1)-)-methyltransferase^1^ | BB0698 | Protein synthesis | Chromosome |
| polynucleotide adenylyltransferase (papS)^1,2^ | BB0706 | Transcription | Chromosome |
| hypothetical protein BB0707^1,2^ | BB0707 | Hypothetical proteins | Chromosome |
| hypothetical protein BB0709^1,2^ | BB0709 | Hypothetical proteins | Chromosome |
| DNA primase (dnaG)^1^ | BB0710 | DNA metabolism | Chromosome |
| **penicillin-binding protein (pbp-2)^1,2^** | **BB0718** | **Cell envelope** | **Chromosome** |
| rod shape-determining protein (mreB-2)^1^ | BB0719 | Cellular processes | Chromosome |
| CDP-diacylglycerol--glycerol-3-phosphate 3-phosphatidyltransferase^2^ | BB0721 | Fatty acid and phospholipid metabolism | Chromosome |
| hypothetical protein BB0722^1,2^ | BB0722 | Hypothetical proteins | Chromosome |
| ATP-binding protein (ylxH-3)^1,2^ | BB0726 | Unknown function | Chromosome |
| hypothetical protein BB0734^1,2^ | BB0734 | Unknown function | Chromosome |
| histidine phosphokinase/phophatase, putative^1,2^ | BB0737 | Regulatory functions | Chromosome |
| hypothetical protein BB0748^2^ | BB0748 | Cellular processes | Chromosome |
| **hypothetical protein BB0761^1^** | **BB0761** | **Cell envelope** | **Chromosome** |
| hypothetical protein BB0765^1,2^ | BB0765 | Hypothetical proteins | Chromosome |
| putative DNA-binding/iron metalloprotein/AP endonuclease^1,2^ | BB0769 | Protein fate | Chromosome |
| hypothetical protein BB0773^1,2^ | BB0773 | Hypothetical proteins | Chromosome |
| hypothetical protein BB0788^1,2^ | BB0788 | Protein synthesis | Chromosome |
| hypothetical protein BB0792^1^ | BB0792 | Hypothetical proteins | Chromosome |
| queuine tRNA-ribosyltransferase^1^ | BB0809 | Protein synthesis | Chromosome |
| pantothenate metabolism flavoprotein (dfp)^1,2^ | BB0812 | Biosynthesis of cofactors, prosthetic groups, and carriers | Chromosome |
| hypothetical protein BB0815^1^ | BB0815 | Protein synthesis | Chromosome |
| cytidylate kinase^1,2^ | BB0819 | Purines, pyrimidines, nucleosides, and nucleotides | Chromosome |
| tRNA delta(2)-isopentenylpyrophosphate transferase^1,2^ | BB0821 | Protein synthesis | Chromosome |
| **hypothetical protein BB0823^1,2^** | **BB0823** | **Cell envelope** | **Chromosome** |
| hypothetical protein BB0826^1^ | BB0826 | Hypothetical proteins | Chromosome |
| phosphomannomutase (cpsG)^1,2^ | BB0835 | Energy metabolism | Chromosome |
| hypothetical protein BB0852^1^ | BB0852 | DNA metabolism | Chromosome |
| hypothetical protein BBA23^1^ | BBA23 | Unclassified | lp54 |
| hypothetical protein BBA31^2^ | BBA31 | Mobile and extrachromosomal element functions | lp54 |
| hypothetical protein BBA38^1,2^ | BBA38 | Mobile and extrachromosomal element functions | lp54 |
| hypothetical protein BBA42^1,2^ | BBA42 | Hypothetical proteins | lp54 |
| hypothetical protein BBA50^1,2^ | BBA50 | Hypothetical proteins | lp54 |
| hypothetical protein BBA55^1,2^ | BBA55 | Hypothetical proteins | lp54 |
| hypothetical protein BBA56^1^ | BBA56 | Hypothetical proteins | lp54 |
| hypothetical protein BBB02^1^ | BBB02 | Hypothetical proteins | cp26 |
| hypothetical protein BBD13^1,2^ | BBD13 | Hypothetical proteins | lp17 |
| hypothetical protein BBD22^1^ | BBD22 | Hypothetical proteins | lp17 |
| hypothetical protein BBG25^1^ | BBG25 | Unknown function | lp28-2 |
| hypothetical protein BBG29^1^ | BBG29 | Mobile and extrachromosomal element functions | lp28-2 |
| hypothetical protein BBG33^2^ | BBG33 | Unclassified | lp28-2 |
| hypothetical protein BBH27^2^ | BBH27 | Unknown function | lp28-3 |
| hypothetical protein BBJ18^1,2^ | BBJ18 | Unknown function | lp38 |
| hypothetical protein BBK22^2^ | BBK22 | Unknown function | lp36 |
| hypothetical protein BBN12^1,2^ | BBN12 | Hypothetical proteins | cp32-9 |
| hypothetical protein BBR06^1^ | BBR06 | Hypothetical proteins | cp32-4 |
| hypothetical protein BBS26^1^ | BBS26 | Unknown function | cp32-3 |
